# Abnormal findings in fetal echocardiography and maternal disease: A cross-sectional study

**DOI:** 10.18502/ijrm.v20i5.11055

**Published:** 2022-06-08

**Authors:** Mohammad Reza Alipour, Hossein Moradi, Seyedeh Mahdieh Namayandeh, Fatemeh Majidpoure, Zohreh Pezeshkpour, Mohammadtaghi Sarebanhasanabadi

**Affiliations:** ^1^Yazd Cardiovascular Research Center, Non-communicable Diseases Research Institute, Shahid Sadoughi University of Medical Sciences, Yazd, Iran.; ^2^Shahid Sadoughi University of Medical Sciences, Yazd, Iran.; ^3^Research and Clinical Center Infertility, Yazd Reproductive Sciences Institute, Shahid Sadoughi University of Medical Sciences, Yazd, Iran.

**Keywords:** Fetus, Echocardiography, Heart defects, Congenital.

## Abstract

**Background:**

Congenital cardiovascular malformation is the most common group of birth defects. Fetal echocardiography is highly sensitive and specific in the diagnosis of congenital heart disease in low- and high-risk populations.

**Objective:**

This study aimed to assess abnormal findings in fetal echocardiography and maternal disease.

**Materials and Methods:**

This cross-sectional study was performed on 114 pregnant women referred to Afshar hospital, Yazd, Iran from October 2016 to March 2017. All pregnant women underwent fetal echocardiography through fetal heart screening protocol, which is administered by the international society of ultrasound in obstetrics and gynecology guidelines. Data collected included referral cause, gestational age, maternal age, gravida, and final diagnosis after an accurate fetal echocardiography.

**Results:**

The mean gestational age was 20 wk. The most common referral cause of pregnant women included diabetes (36.8%), fetal arrhythmia (14%), high maternal age (7%), and echogenic focus on ultrasound (6.1%). The most common postpartum findings were normal (68.4%), cardiac abnormalities (17.6%), and arrhythmias (14%). In postnatal echocardiography, the results were consistent with fetal echocardiography except in 2 women.

**Conclusion:**

This study showed that fetal echocardiography can be used in the early diagnosis and treatment of congenital heart diseases.

## 1. Introduction

Congenital heart disease (CHD) is usually triggered by an abnormal development in the fetal heart structure in early embryonic stage (1). Some abnormalities may occur when the heart splits into 4 chambers and the valves form. Conotruncal disorders or valvular formation anomalies occur during these stages. These defects do not trigger prenatal or dynamic problems (2). Congenital vascular malformations are the most common group of birth defects and affect about 6-8 per thousand newborns (3). According to the previous studies, the prevalence of CHD is 6 times higher than chromosomal abnormalities and 4 times higher than neural tube defects. There are several risk factors involved in these diseases, which are generally divided into 2 categories maternal and fetal (4). In most cases, the cause is unknown; some are genetic, and some are environmental (5). Although in most cases they are multifactorial, several are also associated with chromosomal abnormalities, congenital defects, teratogens, or maternal metabolic disease. The range of lesions varies from asymptomatic to fatal anomalies (6).

Due to the high prevalence of congenital heart defects, fetal echocardiography is an important part of screening during pregnancy to diagnose fetal heart defects and arrhythmias (7). Fetal echocardiography has high sensitivity and specificity in diagnosing CHD in low- and high-risk populations (8). Fetal echocardiography is considered a standard part of pregnancy services (9). Techniques such as fetal heart magnetic resonance imaging and electrocardiogram are performed in only a few facilities due to their high cost and difficulty of access and are used only in research protocols. Fetal cardiac magnetic resonance imaging and fetal electrocardiography alternatives are acceptable procedures in diagnosing fetal atrial and ventricular arrhythmias (10). Although in fetal echocardiography, it is impossible to calculate details such as the p-wave axis or the width of QRS and QT ${}_{\text{C}}$ interval, the type of arrhythmia can only be identified based on changes in Doppler waves (11).

Moreover, in several other cases, such as fetal tachyarrhythmias, it is possible to prevent or treat heart failure by drugs prescribed to the mother. Another application of echocardiography in the treatment of non-autoimmune hydrops associated with fetal heart failure is performed by prescribing high-dose digoxin to the pregnant mother and monthly follow-up (4). Also, with early diagnosis of these diseases, fetal heart interventions can be used to treat these defects. One of the most successful of these interventions is transplacental drug therapy to treat fetal tachyarrhythmias (7).

Therefore, this study aimed to assess abnormal findings observed in fetal heart based on echocardiography and maternal disease.

## 2. Materials and Methods

This cross-sectional study was performed on 114 pregnant women referring to Afshar hospital, Yazd, Iran, from October 2016 and March 2017. Our inclusion criteria were pregnant women with a gestational age of 12 wk referred by gynecologists or radiologists for fetal echocardiography based on the international society of ultrasound in obstetrics and gynecology. All women in the first trimester of pregnancy, having a pregnancy with intrauterine fetal death, threatened with abortion fetuses, and incomplete abortion were excluded.

We intend to initially evaluate the diagnostic value of fetal echocardiography for early diagnosis of congenital diseases and also regarding the fact that some of these diseases are hard to cure. Although the standard time for fetal echocardiography to diagnose cardiac anomalies was before 19 wk of gestation, since several mothers were referred to evaluate diseases that could be medically intervened at any time (including fetal arrhythmia and heart failure), therefore, the referrals that after 19 wk of gestation were also included in the study. A pediatric cardiologist performed all echocardiography to prevent information bias. Participants' information including maternal age, gestational age, number of fetuses, underlying maternal disease, history of drug use or addiction in the mother, family history of CHD, history of infertility or use of assisted reproductive techniques, history of infectious diseases in the mother such as rubella, referral indication as well as final diagnosis after accurate fetal echocardiography was recorded. Echocardiography was performed by Color Doppler Echocardiograph, the Vivid 3 expert model (GE Healthcare, made in the USA), version 2011, and transducer containing crystal sets for 2-dimensional image (3.5 MHz with second harmonic technology). Also, follow-up echocardiography was done during 1 ${}^{\text{st}}$ wk after birth for matching pre and post-natal diagnosis.

### Ethical considerations

The Ethical Committee of Shahid Sadoughi University of Medical Sciences, Yazd, Iran was approved the study proposal (Code: IR.SSU.MEDICINE.REC.1397.149). The oral consent was obtained from all participants.

### Statistical analysis

The qualitative variables were reported as frequency and percentage. Data were analyzed by SPSS19 (IBM Corporation, New York, USA).

## 3. Results

**Figure 1:**
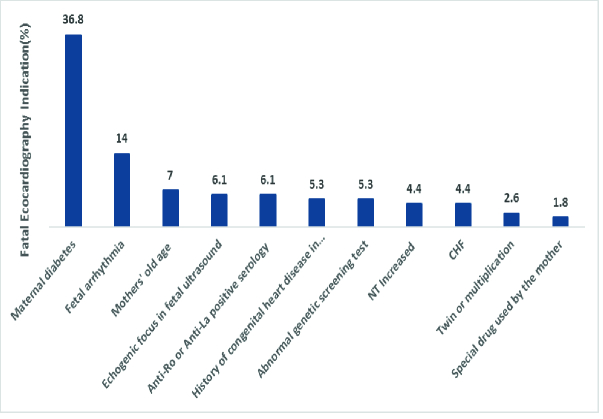
The distribution of the frequency of referral causes of pregnant mothers, IVF: In vitro fertilization, IUI: Intrauterine insemination, NT: Nuchal translucency, CHF: Congestive heart failure.

A total of 114 pregnant women who underwent complete echocardiography and follow-up were included. The mean age of the pregnant women and their mean gestational age were 27 yr and 20 wk, respectively. The most common reason was diabetes (36.8%). While, 14% (n = 16) of referrals were due to fetal arrhythmia [Premature atrial complex (n = 6), bradyarrhythmia (n = 4), tachyarrhythmia (n = 3), and complete heart block (n = 3)]. The results of fetal echocardiography revealed that 68.4% (n = 78) were normal, 17.6% (n = 20) had cardiac anomalies, and 14% (n = 16) had arrhythmia (Figure 1).

**Figure 2:**
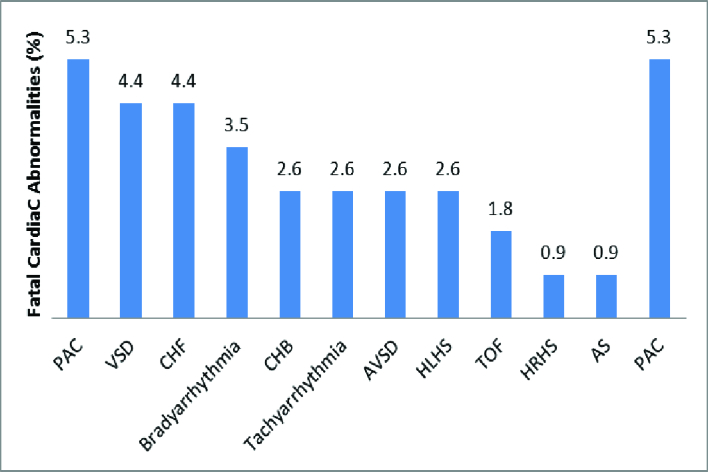
The percentage of different types of cardiac abnormalities detected by echocardiography, PAC: Premature atrial contraction, VSD: Ventricular septal defect, CHF: Congestive heart failure, CHB: Complete heart block, AVSD: Atrioventricular septal defect, HLHS: Hypoplastic left heart syndrome, TOF: Tetralogy of fallot, HRHS: Hypoplastic right heart syndrome, AS: Aortic stenosis.

Figure 2 shows the percentage of different types of cardiac abnormalities detected by echocardiography. The distribution of the frequency percentage of cardiac abnormalities detected by fetal echocardiography in each gestational age interval is reported in the table I, the highest rate found in 16-19 wk of pregnancy (44.4%).

Table II shows the frequency distribution of cardiac abnormalities diagnosed based on the age of pregnant women. The highest rate of these abnormalities was in mothers aged 26-31 yr, which included 16 cases. Out of 42 (36.8%) pregnant women referred due to diabetes, 11 (9.6%) women had type 1 diabetes, and 31 (27.2%) were affected by type 2. On echocardiography 2 (1.8%) women were reported with cardiac abnormalities, both of which were related to type 1 diabetes. Of 5 cases (4.4%) of pregnant women who were referred due to in vitro fertilization, a cardiac abnormality was identified in 1 case (0.9%), whereas 4 other cases (3.5%) were normal. Also, 2 cases (1.8%) of pregnant women were referred due to a history of abortion; in both cases, a cardiac abnormality was observed. Among 6 women (5.3%) with a positive family history, 2 cases (1.8%) had a history of CHD in the mother or relatives and 4 women (3.5%) had a history disease in their previous children or fetuses. Cardiac abnormalities were observed in all 6 cases. Also, 2 cases (1.8%) were referred due to drug use in the mother; one of which (0.9%) was diagnosed with cardiac abnormality (Table III).

In postnatal echocardiography, out of 114 cases, only the results of 2 cases didn't match the prenatal results [2 cases of the small ventricular septal defect (VSD) which missed in fetal echocardiography]. Thus, the sensitivity of fetal echocardiography for early diagnosis of CHD and specificity were determined as 94.7 and 100%, respectively. Out of 114 referred pregnant women, 3 were treated with tachyarrhythmia, 3 with complete heart block, and 5 others with congestive heart failure, all showed signs of improvement (Table III).

**Table 1:** Frequency distribution of cardiac abnormalities detected by fetal echocardiography


**Gestational age (wk)**	**Cardiac abnormalities (%)**
**< 16**	11.2
**16-19**	44.4
**20-23**	2.8
**24-27**	8.3
**28-31**	8.3
**32-35**	16.7
**> 36**	8.3

**Table 2:** Frequency distribution of cardiac abnormalities diagnosed based on the mother's age


	**Fetal heart echo**	
**Mother's age (Yr)**	**Normal**	**Cardiac abnormalities**
	**< 20**	13	2
**20-25**	22	10
**26-31**	23	16
**32-37**	16	8
**38-41**	3	0
**> 42**	1	0
**Total**	78	36
Data presented as number		

**Table 3:** Frequency distribution of echocardiography results based on referal causes of pregnant women


	**Fetal echocardiography**	
**Referral indication**	**Normal**	**Abnormal**
	**Maternal diabetes**	40 (95.2)	2 (4.8)
**Fetal arrhythmia**	0 (0)	16 (100)
**Mothers' old age**	6 (75)	2 (25)
**Echogenic focus in fetal ultrasound**	6 (85.7)	1 (14.3)
**Anti-Ro or Anti-La positive serology **	7 (100)	0 (0)
**IVF, IUI, or history of recurrent miscarriage **	4 (57.1)	3 (42.9)
**History of congenital heart disease in the mother,** **relatives, or previous children**	0 (0)	6 (100)
**Abnormal genetic screening test**	6 (100)	0 (0)
**Increased nuchal translucency **	5 (100)	0 (0)
**Congestive heart failure**	0 (0)	5 (100)
**Twin or multiple pregnancy **	3 (100)	0 (0)
**Special drug used by the mother**	1 (50)	1 (50)
**Total**	78 (68.4)	36 (31.6)
Data presented as n (%). IVF: In vitro fertilization, IUI: Intrauterine insemination		

## 4. Discussion

In this study, of 114 referred pregnant women, the most common reason for fetal echocardiography was diabetes (36.8%), 14% of referrals were due to fetal arrhythmia, and 49.2% were because of other problems. The results of fetal echocardiography revealed that 68.4% of cases were normal, 17.6% had cardiac anomalies, and 14% had abnormal cardiac rhythm.

In fetal echocardiography, we are faced with a host of images that fail to be sequenced, all of which may lead to errors in the correct diagnosis of fetal heart abnormalities or arrhythmias, thus warning us of the need for postnatal follow-up echocardiography (12).

In this study, the prevalence of CHD was 13.1%. However, in a study that had examined 1,200 cases of pregnancy for a year, the incidence of CHD was reported to be 15 per 1,000 births (4). Further, in research, the frequency was detected as 20.3 per 1000 births (4, 13). However, this in other studies has averaged 8 per thousand births, and the AHA guideline reports it as 6-12 per thousand live births. Of course, it must be taken into account our study population was selected based on the international society of ultrasound in obstetrics and gynecology guidelines, while in other similar studies, they were selected from the general population of pregnant women.

In the present study, the mean age of the mothers referred was 26.9 yr, while in another study, it reached 27.6 yr (4). Also, the mean gestational age of mothers in the present study was 19.75 wk. This rate amounted to 20.37 wk in Sharma's probe but 23.1 wk in Barsoom (4, 14). The highest cardiac abnormality diagnosed by fetal echocardiography was 16-19 wk of gestation. In reviewing other literature, the best time for diagnosis was attributed to referral in the second trimester; considering that the gestational age of the mothers referred in the present study was at the range of 12-37 wk, it seems that the age for referral has been late. Due to the need for religious and legal restrictions to terminate the pregnancy, this referral should preferably be before 19 wk. In this study, the most common reason for referral was maternal diabetes, which accounted for 36.8% of the cases. Fetal arrhythmias being 14% and maternal old age of 7% were other reasons for referral of the mothers. Of course, this rate can be justified due to the high prevalence of diabetes in Yazd, Iran, while in Barsoom and co-workers study, aneuploidy risk with 37.2% and echogenic focus with 12.1% were the most causes of fetal echocardiography, and maternal diabetes was identified in only 7.7%. Also, in another survey, the presence of echogenic focus was detected in 48% of cases, an increase in nuchal translucency thickness in 13%, and maternal age in 10% as the reasons for the referral of the mothers. In our study, the most common type of CHD identified was VSD with 4.4%. Also, the most common type of arrhythmia was related to premature atrial complex with 5.3% while in Mottaghi's research, CHD complex with 11.2%, in Sharma's study, VSD with 44.4%, in Nayak's study, endocardial cushion defect with 19.2% and in Chitra's study, VSD with 18.4% were the most common types of CHD reported (4, 13, 15, 16). Comparing the fetal and postnatal echocardiography results in this study, 94.4% sensitivity and 100% specificity were obtained for fetal echocardiography, while in Rakha and co-worker study; sensitivity and specificity were evaluated as 97.03 and 99.07%, respectively (17). Also, Sharma and colleagues reported a complete association between the 2 echocardiography amounting to 68.17% (4). Further, in another study, the sensitivity and specificity of the method were expressed as 85.5 and 100%, respectively (18).

## 5. Conclusion

This study showed that fetal echocardiography is a non-invasive method suitable for early diagnosis of CHDs with high sensitivity and specificity, being appropriate in high-risk and low-risk pregnancies. Also, it could be employed in early diagnosis and treatment of CHD if present or in abortion therapy.

##  Conflict of Interest

There was no conflict of interest in the study.
